# Supplementary material for: Serum albumin is independently associated with higher mortality in adult sickle cell patients: Results of three independent cohorts
Source: PLoS One. 2020 Aug 10;15(8):e0237543. doi: 10.1371/journal.pone.0237543 (PMC7416942; doi:10.1371/journal.pone.0237543)
Supplement: S1 Table — Results are in median (IQR) unless otherwise specified. (DOCX) [file pone.0237543.s001.docx]

**Table S1. Unadjusted correlation between serum albumin and clinical variables in adults with sickle cell disease in test cohorts. Results are in median (IQR) unless otherwise specified.**

|  | | | | | | |
| --- | --- | --- | --- | --- | --- | --- |
|  | Walk-PHaSST | | | CSSCD | | |
|  | N | Results | Correlation coefficient (P) | N | Results | Correlation coefficient (P) |
| Age (year) | 630 | 37 (27-47) | **-0.27 (<0.001)** | 1303 | 27 (22-33) | **-0.14 (<0.001)** |
| Female gender, n (%) | 630 | 336 (53) | -0.07 (0.08) | 1303 | 740 (57) | **-0.06 (0.023)** |
| SS genotype, n (%) | 604 | 466 (77) | 0.02 (0.67) | 1293 | 661 (51) | -0.02 (0.55) |
| Number of severe pains in last year, n (%) | 630 | 2 (0-5) | 0.04 (0.27) |  | -- | -- |
| Chronic transfusion, n (%) | 627 | 74 (12) | 0.04 (0.32) | 1023 | 41 (4) | **-0.09 (0.005)** |
| History of acute chest syndrome, n (%) | 630 | 392 (62) | -0.01 (0.87) |  | -- | -- |
| Leg ulcer, n (%) | 630 | 126 (20) | -0.05 (0.19) | 1277 | 628 (49) | **-0.06 (0.046)** |
| BMI (kg/m^2^) | 619 | 23.6 (21.3-27.1) | -0.04 (0.28) | 1227 | 20.8 (19.0-23.5) | -0.04 (0.13) |
| Hemoglobin (g/dL) | 624 | 9.2 (8.0-10.7) | **0.14 (<0.001)** | 1185 | 9.3 (8.2-11.1) | **0.11 (<0.001)** |
| MCV (fL) | 623 | 90 (82-98) | -0.05 (0.20) | 1182 | 89 (82-96) | **-0.06 (0.045)** |
| White blood cell count (x10^9^/L) | 624 | 9.2 (7.0-11.6) | 0.05 (0.25) | 1185 | 10.4 (8.3-12.7) | **-0.07 (0.011)** |
| Platelet count (x10^9^/L) | 623 | 339 (261-432) | 0.03 (0.45) | 1130 | 375 (285-475) | -0.03 (0.27) |
| Lactate dehydrogenase (U/L) | 590 | 366 (249-553) | **0.13 (<0.001)** | 1275 | 335 (218-495) | **0.06 (0.045)** |
| Reticulocyte count (x10^9^/L) | 593 | 216 (140-317) | **0.11 (0.006)** | 1150 | 250 (162-378) | **-0.09 (0.004)** |
| Serum albumin (g/L) | 630 | 42 (39-44) | -- | 1303 | 44 (42-46) | -- |
| Total bilirubin (mg/dL) | 629 | 2.3 (1.4-3.6) | 0.01 (0.83) | 1294 | 2.2 (1.4-3.6) | **0.08 (0.006)** |
| Alanine aminotransferase (U/L) | 630 | 22 (16-32) | -0.05 (0.22) | 1049 | 20 (12-34) | **-0.10 (0.001)** |
| Aspartate aminotransferase (U/L) | 617 | 38 (27-53) | -0.05 (0.19) | 1286 | 40 (25-67) | **-0.12 (<0.001)** |
| Alkaline phosphatase (U/L) | 630 | 85 (66-113) | **-0.18 (<0.001)** | 1290 | 85 (65-116) | **-0.17 (<0.001)** |
| Creatinine (mg/dL) | 629 | 0.7 (0.6-0.9) | **-0.22 (<0.001)** | 1263 | 0.8 (0.6-1.0) | -0.04 (0.15) |
| eGFR (mL/min/1.73m^2^) | 629 | 129 (101-146) | **0.28 (<0.001)** | 1263 | 134 (106-149) | **0.08 (0.003)** |
| NT-proBNP (pg/mL) | 587 | 68 (30-157) | **-0.26 (<0.001)** | 316 | 66 (22-170) | -0.06 (0.25) |
| Tricuspid regurgitation velocity, m/sec | 563 | 2.5 (2.3-2.8) | **-0.27 (<0.001)** | -- | -- | -- |
