# Supplementary material for: Serum albumin is independently associated with higher mortality in adult sickle cell patients: Results of three independent cohorts
Source: PLoS One. 2020 Aug 10;15(8):e0237543. doi: 10.1371/journal.pone.0237543 (PMC7416942; doi:10.1371/journal.pone.0237543)
Supplement: S2 Table — Results are in median (IQR) unless otherwise specified. (DOCX) [file pone.0237543.s002.docx]

| **Table S2- Unadjusted correlation between serum albumin and sickle cell clinical variables in adults with sickle cell disease in validation cohort (OMG). Results are in median (IQR) unless otherwise specified.** | | | |
| --- | --- | --- | --- |
|  | N | Results | correlation (p) |
| Age (year) | 620 | 32 (23-42) | **-0.27 (<0.001)** |
| Female gender, n (%) | 620 | 339 (55) | -0.07 (0.09) |
| SS genotype, n (%) | 613 | 509 (83) | **-0.08 (0.046)** |
| Hospitalizations for severe pain in last year, n (%)  0-1  2-4  >4 | 559 | 319 (57)  149 (27)  91 (16) | -0.04 (0.39) |
| Chronic transfusion, n (%) | 573 | 36 (6) | -0.01 (0.78) |
| History of acute chest syndrome, n (%) | 579 | 424 (73) | -0.05 (0.23) |
| Leg ulcer, n (%) | 571 | 126 (22) | **-0.15 (<0.001)** |
| BMI(kg/m^2^) | 433 | 23.5 (20.7-28.1) | -0.02 (0.65) |
| Hemoglobin (g/dL) | 536 | 8.6 (7.7-10.0) | **0.22 (<0.001)** |
| Current MCV (fL) | 536 | 91 (84-99) | **-0.09 (0.044)** |
| Current WBC(x10^9^/L) | 536 | 10.7 (8.3-13.7) | 0.07 (0.10) |
| Current Platelet(x10^9^/L) | 534 | 395 (298-478) | -0.07 (0.13) |
| Lactate dehydrogenase (U/L) | 504 | 231 (166-334) | **-0.09 (0.045)** |
| Reticulocyte count (x10^9^/L) | 455 | 254 (180-347) | 0.04 (0.35) |
| Serum albumin (g/dL) | 620 | 42 (39-45) | -- |
| Total bilirubin (mg/dL) | 611 | 2.3 (1.3-3.7) | 0.06 (0.17) |
| Alanine aminotransferase (U/L) | 615 | 24 (16-35) | **-0.15 (<0.001)** |
| Aspartate aminotransferase (U/L) | 614 | 38 (27-54) | **-0.16 (<0.001)** |
| Alkaline phosphatase (U/L) | 610 | 91 (72-121) | **-0.16 (<0.001)** |
| Creatinine (mg/dL) | 616 | 0.7 (0.6-0.9) | **-0.14 (<0.001)** |
| eGFR (mL/min/1.73m^2^) | 616 | 138 (112-154) | **0.29 (<0.001)** |
| NT-proBNP (pg/mL) | 87 | 81 (38-246) | -0.13 (0.23) |
| Tricuspid regurgitation velocity (m/sec) | 106 | 2.2 (1.8-2.6) | **-0.30 (0.002)** |
