# Supplementary material for: Serum albumin is independently associated with higher mortality in adult sickle cell patients: Results of three independent cohorts
Source: PLoS One. 2020 Aug 10;15(8):e0237543. doi: 10.1371/journal.pone.0237543 (PMC7416942; doi:10.1371/journal.pone.0237543)
Supplement: Attachment — Submitted filename: Response to Reviewers.docx [file pone.0237543.s003.docx]

PONE-D-20-14109

Dear Dr. Hodges;

We appreciate your and review comments on our manuscript. Hereby, we are responding to comments and addressing changes in the manuscript:

**Comment:** The conclusion could have been bolstered by more references to the literature on albumin synthesis rates in children with acute-phase protein responses to infection in edematous and non-edematous protein-energy malnutrition. The speculation that hepatic synthetic rates may be affected because of the association of low albumin with liver enzyme elevation raised concern in the concluding paragraph which should be re-phrased.

**Response:** We modified the discussion and conclusion to reflect the reviewer’s comments.

**Comment:** In your Data Availability statement, you have not specified where the minimal data set underlying the results described in your manuscript can be found. PLOS defines a study's minimal data set as the underlying data used to reach the conclusions drawn in the manuscript and any additional data required to replicate the reported study findings in their entirety. All PLOS journals require that the minimal data set be made fully available. For more information about our data policy, please see <http://journals.plos.org/plosone/s/data-availability>.

Upon re-submitting your revised manuscript, please upload your study’s minimal underlying data set as either Supporting Information files or to a stable, public repository and include the relevant URLs, DOIs, or accession numbers within your revised cover letter. For a list of acceptable repositories, please see <http://journals.plos.org/plosone/s/data-availability#loc-recommended-repositories>. Any potentially identifying patient information must be fully anonymized.

Important: If there are ethical or legal restrictions to sharing your data publicly, please explain these restrictions in detail. Please see our guidelines for more information on what we consider unacceptable restrictions to publicly sharing data: <http://journals.plos.org/plosone/s/data-availability#loc-unacceptable-data-access-restrictions>. Note that it is not acceptable for the authors to be the sole named individuals responsible for ensuring data access.

We will update your Data Availability statement to reflect the information you provide in your cover letter.

**Response:** The current manuscript is using three different databases. Data from one of these cohort (CSSCD) was received through an agreement with NHLBI/BioLincc. This agreement prohibits investigator form any further data sharing. However, other investigators can apply directly to BioLincc for their own access to the data. The remaining two cohorts are governed by academic institutional IRBs which have their own restrictions on sharing. However, both of the other cohorts are available for access via dbGaP. So, regretfully we are unable to comply within the legal terms of our data use agreement.

**Comment:** We note that you have included the phrase “data not shown” in your manuscript. Unfortunately, this does not meet our data sharing requirements. PLOS does not permit references to inaccessible data. We require that authors provide all relevant data within the paper, Supporting Information files, or in an acceptable, public repository. Please add a citation to support this phrase or upload the data that corresponds with these findings to a stable repository (such as Figshare or Dryad) and provide and URLs, DOIs, or accession numbers that may be used to access these data. Or, if the data are not a core part of the research being presented in your study, we ask that you remove the phrase that refers to these data.

**Response:** This sentence was changed to:

*“We did not measure these proteins in all cohorts but an analysis in the Walk-PHaSST cohort showed that serum albumin and haptoglobin are not correlated (r = 0.06, P = 0.17).”*

**Reviewer #1 comment:** This paper reports on the outcome of analysis on 3 large retrospective cohort of subjects with Sickle Cell Disease. The hypotheses, and justification were clear. The results were clear. Albumin as a negative acute phase protein is a biomarker for worse outcome and this is consistent across a range of clinical models. The conclusion could have been bolstered by reference to the literature where albumin synthesis rates were measured in vivo. So in a model of severe malnutrition in children albumin concentration were modulated by catabolic rates rather than synthesis rates. In fact children with severe malnutrition were able to synthesize a range of positive acute phase proteins at fast enough rate in response to infections. So Sickle cell as an example of inflammatory state may behave similarly. (Morlese JF, Forrester T, Badaloo A, Del Rosario M, Frazer M, Jahoor F. Albumin kinetics in edematous and nonedematous protein-energy malnourished children. Am J Clin Nutr. 1996 Dec;64(6):952-9. doi: 10.1093/ajcn/64.6.952. PMID: 8942422 & Reid, M., Badaloo, A., Forrester, T., Morlese, J.F., Heird, W.C. & Jahoor, F. (2002) The acute-phase protein response to infection in edematous and nonedematous protein-energy malnutrition. Am J Clin Nutr, 76, 1409-1415.). While the authors speculated in the manuscript that hepatic synthetic rates may be affected because of the association of low albumin with liver enzyme elevation this may not be the case. Within that context I am little concerned about the wording of the concluding paragraph which at a casual glance may seem to be advocating supplemental albumin treatment in SCD and perhaps a clear definite statement that this is not their recommendation should be made

**Response:** We cited these references in discussion as:

*“Studies in children with severe malnutrition have shown that plasma albumin concentration was modulated by catabolic rates rather than synthesis rates[19, 20]. SCD patient are in a state of hyper-inflammation which could lead to faster catabolic rates.”*

Also, we changed the conclusion to:

*“There are existing controversies to consider albumin as a supplementary treatment for liver and renal failure as well as volume resuscitation in sepsis [37]. Our findings support the importance of plasma albumin measure and potential benefit of boosting plasma levels of albumin in SCD, especially in SCD endemic countries with high frequency of protein-calorie malnutrition.”*

 Sincerely yours,

Mehdi Nouraie, MD, PhD
