# Supplementary material for: Serum albumin is independently associated with higher mortality in adult sickle cell patients: Results of three independent cohorts
Source: PLoS One. 2020 Aug 10;15(8):e0237543. doi: 10.1371/journal.pone.0237543 (PMC7416942; doi:10.1371/journal.pone.0237543)
Supplement: Attachment — Submitted filename: Response to Reviewers_3.docx [file pone.0237543.s004.docx]

Dear Dr. Hodges

Authors appreciate your and reviewers’ invaluable new comments on our manuscript. Hereby, we are addressing the comments and changes in the new version.

Editor Review

Please address the inter-study variability, possible causes and study limitations

Response: We added this statement to study limitation:

*“Serum albumin was comparable between all three cohorts whereas inter-study variation exists in some other clinical variables. This variability could be due to random variation or different patient characteristics including age, disease severity, genetic structure, disease modifying treatments (including hydroxyurea and chronic transfusion) and comorbidities (as well as their treatment). Other factors that can cause inter-study variability are different birth cohorts and time period between CSSCD and the two other studies, differences in the study protocol and laboratory measurements. These differences could limit the generalizability of our findings to any new sickle cell cohort.”*

Reviewer #3 comments:

This paper presents the results for 3 different SSD studies. It was found that low serum albumin was associated with higher mortality across all 3 studies. However, the paper also looks at predictors of low serum albumin in Tables 1 and 2 of the manuscript. There is a large amount of inter-study variability which is not well accounted for. It seems possible that there are significant differences in the enrolled populations of the 3 studies and that these differences may impact the predictors relationship with serum albumin.

See below for specific comments.

1.1 Specific Comments

1. The term “predicts” in the title is misleading. Many people will interpret the claim that “Serum albumin independently predicts higher mortality” as a statement of that serum albumin has a high predictive value (sensitivity, specificity, AUC, etc) with respect to mortality. However, what is shown is that low serum corresponds to a high hazard ratio. I think “Serum albumin is independently associated with higher mortality” is a more appropriate phrase.

Response: We changed the title to:

*“Serum albumin is independently associated with higher mortality in adult sickle cell patients: Results of three independent cohorts”*

2. You mentioned you tested serum albumin for normality. Do you mean the residuals from the individual fitted linear models are normal? Serum albumin should not be normal on its own, unless the predictor variables are all insignificant. The residuals are what we expect to be normal. In point of fact, when the sample size is large the ordinary least squares estimates are asymptotically normal even if the residuals are non-normal. The sample sizes in this study are large enough that I think the results are valid even if the residuals are not normal.

Response: We removed this statement from the statistical analysis as it did not impact our analysis.

3. The false discovery rate adjustment method and procedure should be stated.

Response:

We added this statement in statistical method:

*“We adjusted for multiple hypotheses testing using Simes method [17]”.*

4. The results of table 1 and 2 make drawing conclusions about associations between serum albumin and sickle cell clinical variables challenging. Many of the variables change signs, even significantly, from study to study. For example ARC beta is significant in the WALK study at 0.03. For CCSSD it is significant at -0.02. Then it is insignificant at 0.001 for OMG. There are many examples of this behavior. Inter-study variability of beta and even significance is to expected; however, when a variable is significant and changes signs from study to study

represents a failure in validation. A disclaimer or a summary across all 3 studies of which variables of interest exhibited consistent behavior would help a reader understand how to better interpret Tables 1 and 2.

Response: We added this statement at the end of first result section:

*“In both Walk-PHaSST and OMG cohorts, higher eGFR was consistently associated with higher serum albumin whereas in CSSCD and Walk-PHaSST cohorts, higher LDH and lower alkaline phosphatase were associated with higher serum albumin.”*

5. Is there any insight into why there is such a significant amount of inter-study variability. Certainly CCSSCD, which ended in 1988, might be difficult to compare to patients collected after the year 2000. Are there differences in Age, hemoglobin, or other variables appearing in tables 1 and 2 between the 3 studies? Perhaps the populations at enrollment are significantly different. If so multivariate models that include the variables in table 1 and 2 might be more consistent.

Response: We added some statements to describe inter-study variability (please see the response to editor comment). In addition, tables 1 and 2 are presenting age and hemoglobin adjusted correlations to partially adjust for baseline differences between three cohorts.

6. In Tables 1 and 2 it is stated that p<0.015 and <0.001 are significant after FDR. However, there are rows in bold with p-values greater than these amounts.

Response: We removed the bold marks to prevent any confusion in these tables.

7. In the discussion it is stated that “ Finally, we also did 203 not correct for multiple testing.” However, in each table the FDR rate is listed. FDR is a multiplicity adjustment method. This is confusing was and FDR used or not?

Response: It was removed from discussion

Sincerely,

Mehdi Nouraie, MD, PhD

Associate Professor of Medicine

University of Pittsburgh
